# Who Multi-Tasks and Why? Multi-Tasking Ability, Perceived Multi-Tasking Ability, Impulsivity, and Sensation Seeking

**DOI:** 10.1371/journal.pone.0054402

**Published:** 2013-01-23

**Authors:** David M. Sanbonmatsu, David L. Strayer, Nathan Medeiros-Ward, Jason M. Watson

**Affiliations:** Department of Psychology, University of Utah, Salt Lake City, Utah, United States of America; Cardiff University, United Kingdom

## Abstract

The present study examined the relationship between personality and individual differences in multi-tasking ability. Participants enrolled at the University of Utah completed measures of multi-tasking activity, perceived multi-tasking ability, impulsivity, and sensation seeking. In addition, they performed the Operation Span in order to assess their executive control and actual multi-tasking ability. The findings indicate that the persons who are most capable of multi-tasking effectively are not the persons who are most likely to engage in multiple tasks simultaneously. To the contrary, multi-tasking activity as measured by the Media Multitasking Inventory and self-reported cell phone usage while driving were negatively correlated with *actual* multi-tasking ability. Multi-tasking was positively correlated with participants’ *perceived* ability to multi-task ability which was found to be significantly inflated. Participants with a strong approach orientation and a weak avoidance orientation – high levels of impulsivity and sensation seeking – reported greater multi-tasking behavior. Finally, the findings suggest that people often engage in multi-tasking because they are less able to block out distractions and focus on a singular task. Participants with less executive control - low scorers on the Operation Span task and persons high in impulsivity - tended to report higher levels of multi-tasking activity.

## Introduction

People are not always content doing one thing at a time. Frequently, they multi-task, that is, they engage in multiple tasks aimed at attaining multiple goals simultaneously. Multi-tasking involves concurrent performance of two or more functionally independent tasks with each of the tasks having unique goals involving distinct stimuli (or stimulus attributes), mental transformation, and response outputs. Although multi-tasking is commonplace, relatively little is known about when and why people perform more than one attention-demanding task at a time. Related to this, little is known about who is most likely to multi-task. Our research represents an initial examination of important factors contributing to multi-tasking behavior. We investigated both the predictors of general multi-tasking and the predictors of a specific and potentially dangerous form of multi-tasking – the usage of cellular communications while driving. Decision theory suggests that people should multi-task when they are good at it and expect to benefit from it. However, we hypothesize that there are important motivations and processes contributing to multi-tasking that have little to do with people’s proficiencies.

Multi-tasking enables people to achieve more goals and to experience more activities. However, engaging in multiple attention demanding tasks simultaneously may be cognitively and physically taxing. Moreover, performance on individual tasks may suffer such that errors are made and overall productivity is diminished. Research on decision making [1]–[3] indicates that the willingness to multi-task should be contingent, in part, on the expected outcomes or consequences. Generally, the people who should be most likely to engage in multiple tasks are those who are good at multi-tasking and who expect the highest rewards and lowest costs. The most effective and efficient multi-taskers should be those who are able to exercise a high level of executive control. Models of executive attention highlight the role of the frontally mediated capacity to maintain task goals and to avoid conflicting distractions [4], [5]. Executive attention is central to multi-tasking because the information and goals relevant to one task must be actively maintained while other tasks are performed. Additionally, when task switching occurs, interference from the representations and stimuli associated with the off task must be minimized. A simple but elegant measure of working memory and executive functioning is the Operation Span (OSPAN) task developed by Engle [6]. The OSPAN task is actually two distinct tasks (memory and math) that are performed concurrently, have distinct stimuli (letters and numbers), have different mental transformations (memorization and arithmetic), have different response outputs (memory recall accuracy and math verification accuracy) that are scored independently (i.e., there is a score for memory performance and a score for math performance and the two scores are independent). That is, the OSPAN task is a classic example of multi-tasking wherein people must simultaneously attempt to perform two independent tasks that compete for limited capacity attention [7].

The notion that people multi-task because they are good at it is challenged by the important work of Ophir, Nass, and Wagner [8] who examined the cognitive abilities of chronic multi-taskers. These researchers developed the Media Survey Questionnaire to measure media related multi-tasking and to identify individuals who frequently engage in multiple tasks concurrently. Ophir et al. [8] found that persons who frequently multi-task actually exhibited greater switching costs while performing dual tasks than infrequent multi-taskers. Moreover, chronically high multi-taskers were more readily distracted by both irrelevant external stimuli and recently activated internal representations during singular task performance. Thus, research suggests that the persons who most frequently multi-task may be those who are the least cognitively equipped to effectively carry out multiple tasks simultaneously.

A more proximal determinant of multi-tasking may be the perceived ability to multi-task. The goals that people pursue and the tasks that they undertake are heavily influenced by their conceptions of their traits and their abilities. When individuals believe that they are capable of multi-tasking successfully, they should be more apt to take on multiple tasks simultaneously. Interestingly, self-conceptions of multi-tasking ability and actual multi-tasking ability may not always work hand in hand to influence decision making. In many behavioral domains, beliefs about the self have been found to be only weakly correlated with actual abilities and traits [9]. This disconnect exists, in part, because people overestimate the favorableness of their personal qualities. For example, research has shown that most people perceive themselves to be more physically attractive than average [10], better drivers than average [11], and better leaders than average [12] despite the obvious truth that most people are average on these dimensions. These findings suggest that people may generally overestimate their ability to multi-task relative to others and that the persons who may be most willing to engage in multiple attention demanding tasks are those who are the most overconfident about their capabilities.

Generally, multi-tasking will entail greater potential rewards and greater potential losses than engagement in a singular task. Individuals, of course, vary significantly in their chronic disposition toward rewards vs. punishments [13]. Persons with a strong approach orientation, that is, a strong reward or gain focused motivational orientation, may be especially enticed to take on multiple tasks because of the high potential rewards. In contrast, persons who are avoidance oriented, that is, who are risk averse and sensitive to losses or punishments, may be more inclined to focus on a singular task rather than multi-task because of the higher potential losses and greater effort associated with trying to do more.

One personality trait that has shown to be been strongly associated with the approach and avoidance orientations that may affect the willingness to multi-task is impulsivity. Impulsivity is a complex construct that is commonly defined a “as a predisposition toward rapid, unplanned reactions to internal or external stimuli without regard to the negative consequences of these reactions” [14]. Studies have shown that impulsive individuals are generally more reward oriented [15], [16] and more responsive to the goals and positive outcomes that are salient in a context than individuals low in impulsivity [17], [18]. At the same time, impulsive individuals are more apt to engage in risky behavior [19], [20], and, hence, may be less sensitive to the potential losses or costs of taking on multiple activities.

One of the important rewards that may motivate people to multi-task is the stimulation afforded by multiple task engagement. People may often choose to multi-task because it is more interesting and challenging, and less boring than performing a singular task. In some instances, they may take on several tasks for the sheer enjoyment of it, even if their overall productivity suffers. Thus, workers commonly listen to music or news while performing a boring job even though it may be distracting and detrimental to their performance. A personality trait that may be associated with multi-tasking because of the stimulation that multiple tasks afford is sensation seeking. Zuckerman [21] characterizes sensation seeking as “…a trait defined by the seeking of varied, novel, complex, and intense sensations and experiences and the willingness to take physical, social, legal, and financial risks for the sake of such experiences.” High sensation seekers may be especially apt to multi-task for the sake of the more varied and complex sensations that are afforded by multiple vs. singular tasks [22], [23]. Moreover, because they are less averse to losses [24], [25], they may be more likely than low sensation seekers to risk the costs of multi-tasking in order to heighten the enjoyableness of their experience.

In some instances, people may multi-task despite the potential losses of doing more because they are unable to focus on a singular task. Often, engagement in secondary tasks or routines is activated by the context, specifically by current goals and the stimuli that are present. People may multi-task in these situations because they are unable to block out distractions and focus on their primary endeavor. Individuals with deficits in executive functioning may be especially apt to multi-task because of an inability to inhibit secondary task engagement. Recent work by Cain and Mitroff [26] also indicates that some individuals maintain a wider attentional scope that contributes to distraction and involvement in secondary task processing.

Interestingly, the persons who are best able to multi-task may also be the persons who are best able to not multi-task. Individuals with a high level of executive control should be able to minimize the distractions and goal conflicts that are disruptive to task switching and multi-task performance. At the same time, they should be able to minimize the distractions and competing goals that diminish singular task focus and that contribute to secondary task involvement. This suggests that measures of executive functioning such as OSPAN task performance could be associated with greater multi-tasking ability and with lower multi-tasking activity.

Impulsivity has been associated with lower executive functioning [27], [28] and reduced behavioral inhibition [29]. This suggests that highly impulsive individuals may have a diminished capacity to block out distractions and focus on a primary task than individuals low in impulsivity. Thus, impulsive individuals may take on multiple tasks not only because they are more strongly attracted to the rewards afforded by multi-tasking but also because they are less able to inhibit secondary task engagement.

In the present study, we attempted to understand why people multi-task by examining who tends to multi-task. Participants in our study completed the Media Survey Questionnaire developed by Ophir et al. [8]. Responses to the questionnaire were used to calculate the media multi-tasking index, a measure of multi-tasking in the media context [8]. Participants also reported the frequency with which they engaged in a particular multi-tasking activity – the usage of a cell phone while driving. At any daylight hour, over 10% of drivers on U.S. roadways are estimated to talk on their cell phones [30]. Research has shown that that driving performance is significantly degraded by cell phone conversations [31], [32]. In fact, the National Safety Council [33] estimates that a minimum of 24% of all accidents and fatalities on U.S. highways are caused by distracted drivers. Thus, our study examined important predictors of both general multi-tasking and a specific, socially-relevant form of multi-tasking.

The research of Ophir, et al. [8] investigated the association between multi-tasking activity and cognitive abilities that are likely to be predictive of multi-tasking, such as task switching. Our study examined more directly the extent to which multi-tasking *ability* predicts multi-tasking *activity* by having participants engage in the OSPAN task. Recall that the OSPAN task requires participants to simultaneously perform two independent tasks that compete for limited capacity attention and performance on the OSPAN task has been shown to predict individual differences in real-world multitasking ability [7]. We tested whether the individuals who have the executive control necessary to perform multiple tasks effectively are more apt to multi-task than persons lacking this control.

Participants’ perceived ability to multi-task was also measured. We were interested in whether people who believe they are good as opposed to bad at multi-tasking are generally more likely to multi-task and whether they are more apt to talk on a cell phone while driving. A secondary aim was to examine whether multi-tasking is a domain in which the correspondence between perceived and actual ability is limited, and in which people are overconfident.

Our study also examined whether the strong approach orientation and weak inhibitions of impulsive individuals are associated with more frequent multi-tasking behavior. Participants completed the 11^th^ version of the Barrett Impulsivity Scale [34], which measures general impulsivity and three subcomponents: motor impulsiveness - acting without thinking; attentional impulsiveness – that inability to focus attention or concentrate; and non-planning impulsiveness – a lack of future thinking or forethought. We anticipated that motor and attentional impulsiveness might be particularly highly associated with multi-tasking.

Finally, the Sensation Seeking Scale (SSS-V) [35] was administered to examine whether sensation seekers are more apt to multi-task. This is a multidimensional measure consisting of four interrelated subscales of boredom susceptibility - an aversion to repetitive or boring tasks or people, disinhibition – seeking release or disinhibited social behavior, experience seeking – the pursuit of an unconventional lifestyle, and thrill and adventure seeking. We anticipated that risk taking for the sake of varied sensations and experiences would be associated with greater multi-tasking activity.

## Methods

Three hundred and ten undergraduates (176 female and 134 male) provided informed consent before participating in the University of Utah IRB approved study for extra course credit. Participants ranged in age from 18 to 44, with a median age of 21 (*SD* = 4.7).

Participants completed a series of questionnaires in the context of a study of driving and driving attitudes. The first set of measures assessed their level of cell phone use while driving. Participants indicated “how often do you use your cell phone while driving?” on a 5 point scale anchored by *never/rarely when I drive* and *every time I drive*. They were also asked to report the percentage of the time they are on the phone while driving, if they use their cell phone while driving.

The next set of measures assessed participants’ beliefs about their multi-tasking abilities. Participants ranked their multi-tasking ability relative to that of other college students on a percentage scale on which 0 indicated *I’m at the very bottom*, 50 indicated *I’m exactly average*, and 100 indicated *I’m at the very top*. They also ranked their abilities relative to other adults in the general population on the same percentage scale. Finally, participants reported “how much difficulty do you have performing multiple tasks simultaneously” relative to other college students on a 5 point scale anchored by *much less difficulty than average* and *much more difficulty than average.* The self-assessments of multi-tasking ability were followed by the two personality scales and the Media Use Questionnaire.

### Barratt Impulsivity Scale (BIS)

We administered the 11^th^ version of the BIS – a 30 item self-report instrument designed to assess general impulsiveness [34]. The 11^th^ version consists of 3 subscales measuring attentional impulsiveness, motor impulsiveness, and non-planning impulsiveness (see [36]–[38] for validation of the three subtrait structure). The scale has been shown to have strong internal consistency and reliability, and is probably the most widely used measure of impulsiveness in research and clinical settings [39].

### Sensation Seeking Scale (SSS)

The SSS is a self-report instrument designed to measure the personality construct of sensation seeking [23]. The revised SSS-V [35] consists of four 10 item subscales of boredom susceptibility, disinhibition, experience seeking, and thrill and adventure seeking, and is reported to have good psychometric properties [40].

### Media Use Questionnaire

The questionnaire of multi-media use developed by Ophir, Nass, and Wagner [8] assesses the time spent using 12 different forms of media: computer based applications such as word processing, web surfing, print media, television, computer based video, music, nonmusic audio, video or computer games, phone voice calls, instant messaging, SMS (text messaging), and email. Respondents report the total number of hours they spent using each medium. In addition, they indicated the extent to which they used each of the other types of media while engaging each primary medium by responding *most of the time*, *some of the time, a little of the time,* or *never*. Following Ophir et al. [8], text messaging was not included as a possible primary medium because it could not be accurately described in terms of hours of use. However, it was still assessed as a secondary activity that could be engaged during the use of a primary medium.

### Operation Span Task (OSPAN)

After completing the questionnaires, participants performed an automated version of the Operation Span (OSPAN) task [41]. Participants were asked to remember a series of 2–5 letters that were interspersed with 12 math problems in which an equation and possible solution were presented for verification. They indicated whether the solutions to the math problems were true or false and recalled the letters in the order that they were presented. For example, in one sequence, participants were presented with “is (3/1) − 1 = 2?” followed by “f” followed by “is (2 * 2) +1 = 4?” followed by “k” followed by a recall probe. Participants should have answered “true” and “false” to the math problems when they were presented and recalled “f” and “k” in the order that they were presented when probed. Trials were pseudorandomized such that participants were unable to predict the set size of upcoming equation–letter pairs. Participants were given points equal to the set size when all of the letters in that set were recalled correctly in serial order (i.e., an absolute span score). Math accuracy was also tracked, and feedback was provided to participants during the task. This feedback was intended to keep problem-solving accuracy above 85% and to encourage participants’ compliance with the dual-task math/memory instructions of the OSPAN task.

## Results

The OSPAN task served as our measure of multi-tasking ability. As noted above, the OSPAN task is actually two distinct tasks (memory and math) that are performed concurrently, have distinct stimuli (letters and numbers), have different mental transformations (memorization and arithmetic), have different response outputs (memory recall accuracy and math verification accuracy) that are scored independently. Following Unsworth et al. [41], 32 participants who failed to correctly verify at least 80 percent of the math problems were excluded from the analysis (final n = 277; 156 males and 121 females). The number of memory words recalled in the correct order were summed to determine the absolute OSPAN task score. This is the measure most commonly used in the literature [41] and the measure that was used in the primary analyses. The absolute score was highly correlated with the total score, *r*(276) = .88, *p*<.01, which sums all of the words correctly recalled in serial order. The mean absolute score was 44 with a standard deviation of 16, and the mean total score was 59 with a standard deviation of 13.

Participants’ ranking of their multi-tasking ability relative to that of other college students was highly correlated with their ranking relative to adults in the general population, *r*(275) = .91, *p*<.01, and their estimation of their difficulty in performing multiple tasks simultaneously which was reversed scored, *r*(275) = .72, *p*<.01. Their rankings of their multi-tasking ability relative to that of adults in the general population was also highly correlated with their estimation of their difficulty in performing multiple tasks simultaneously, *r*(275) = .70, *p*<.01. Thus, there was a high degree of convergence between the three measures of perceived multi-tasking ability.

The two percentage estimates of relative ability were averaged to create the primary measure of perceived multi-tasking ability used in the analyses. The mean percentage estimate of multi-tasking across all participants was 63.0 (*SD = *19). A score of 50 on the percentage estimate was “exactly average”. A comparison of the mean percentage estimate with 50 indicated that participants’ generally estimated their multi-tasking ability to be significantly higher than average, *t*(276) = 11.4, *p*<.01. Fifty-four participants estimated that their ability was below average, 30 estimated that they were exactly average, and 193 estimated that they were above average. Significantly more participants estimated that their multi-tasking ability was better than average than would be expected by chance (binomial test; *p*<.01). Thus, participants in our study substantially overestimated their ability to multi-task relative to others.

The inflated estimations relative to average were not the only indicators that participants’ perceptions of their multi-tasking ability were poorly grounded in reality. OSPAN task performance was not significantly correlated with perceived multi-tasking ability, *r*(275) = .08. Hence, participants who believed they were more capable than others of performing multiple tasks simultaneously were not generally better at multi-tasking, as measured by the OSPAN task, than participants who assessed their ability more modestly.

An index of media multi-tasking (MMI) was derived from the responses to the media use questionnaire using the following formula developed by Ophir, Nass, and Wagner [8]:
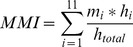



In the formula, *h_i_* is the number of hours per week reportedly spent using primary medium *i* and *h_total_* is the total number of hours per week spent with all primary media. *m_i_* was based on participants’ estimations of time spent on other media activities while engaged with a primary medium. Numeric values were assigned to participants’ estimations as follows: 1 was assigned to *most of the time,*.67 *to some of the time,*.33 to *a little of the time*, and 0 to *never*. The sum of the estimated activities was the *m_i_*. To account for the amount of time spent engaging in the primary medium or activity, the MMI adjusts by dividing the sum of the activity by *h_total_*. The mean level of multi-tasking activity reported on the MMI was 3.99 with a standard deviation of 1.80.

Participants reported the frequency with which they use their cell phones while driving and the percentage of the time they are on the phone while driving. The mean reported frequency of cell phone use while driving was 2.08 on the 5 point scale with a standard deviation of.97. The reported percentage of time on the phone while driving was 13.3 with a standard deviation of 17.3. The reported frequency of cell phone use while driving was strongly correlated with the reported percentage of the time on the phone while driving, *r*(275) = .61, *p*<.01. Because the estimated percentage of time on the cell phone while driving was ostensibly limited to participants who used their cell phone while driving and because of the problems with the numerical figures reported by some participants the first and more inclusive question (the reported frequency of cell phone use while driving) was used in the primary analysis. In reporting the percentage of the time spent on the phone while driving, 70 participants provided figures in decimals (e.g.,.25%). Rather than assuming that these were estimations ranging from 0 to 1% of the time while driving, we assumed that these participants failed to recognize that the estimates were in percentages. Consequently, we ignored the decimals in these participants’ figures. Treating these estimations as whole numbers appears to be correct because it increased the correlation between participants’ percentage estimates and scale estimates of time spent on the phone while driving from *r = *.50 to *r* = .61.

Media related multi-tasking as measured by the MMI and the reported frequency of cell phone use while driving were positively correlated, *r*(275) = .20, *p*<.01. This gives credence to the assumption that both are measures of multi-tasking activity and the willingness to multi-task. A series of correlations were calculated to examine the linkage between multi-tasking activity as reflected by the MMI and cell phone use while driving, and the potential predictors of and contributors to multi-tasking. These correlations are presented in Table 1.

**Table 1:** Correlations between multi-tasking activity, multi-tasking ability, perceived multi-tasking ability, impulsivity, and sensation seeking.

Measure	Subcategory	Multi-tasking Activity (MMI)	Cell Phone Use While Driving
Multi-tasking ability (OSPAN)		−.19**	−.15*
Perceived multi-tasking ability		.19**	.15*
Impulsivity (BIS 11)		.14*	.00
	Attentional impulsiveness	.14*	−.03
	Motor impulsiveness	.14*	.06
	Non planning impulsiveness	.09	−.02
Sensation Seeking (SSS)		.12*	.13*
	Boredom susceptibility	.06	.02
	Disinhibition	.19**	.20**
	Thrill & adventure seeking	.03	.11
	Experience seeking	.04	−.01

N = 277.

* Significant at .05 level.

** Significant at .01 level.

Multi-tasking ability as measured by the OSPAN task was not positively correlated with multi-tasking activity or cell phone use while driving. In fact, multi-tasking activity as reflected by the MMI and cell phone use while driving were significantly negatively correlated with OSPAN task performance. In contrast, multi-tasking and cell phone use while driving were positively correlated with *perceived* multi-tasking ability. Hence, people who believe that they are good as opposed to bad at multi-tasking are more likely to engage in multiple tasks simultaneously and to use a cell phone while driving.

Multi-tasking activity was significantly correlated with impulsivity as measured by the BIS 11. Thus, participants high in impulsivity reported greater multi-tasking than participants low in impulsivity. An examination of the subscale responses revealed that multi-tasking activity was significantly correlated with both attentional and motor impulsivity. Impulsivity was not significantly related to reported cell phone usage while driving.

Finally, multi-tasking activity was significantly correlated with sensation seeking as measured by the SSS. Sensation seeking was similarly positively correlated with cell phone usage while driving. Both multi-tasking activity and cell phone use while driving were associated with the sensation seeking component of disinhibition.

A companion analysis contrasted participants scoring in the upper or lower quartiles in multi-tasking activity. These data are reported in Table 2. Participants in the upper quartile on the MMI scored significantly lower on the OSPAN task and significantly higher in perceived ability, attentional and non-planning components of impulsivity, and the disinhibition component of sensation seeking than participants in the lower quartile on the MMI. In each case, the difference between groups was significant (*p*<.05) and reflected a “medium” effect size (e.g., between.4 and.5) [42].

**Table 2:** Means and standard deviations (in parentheses) for multi-tasking ability (OSPAN), perceived multi-tasking ability, impulsivity, and sensation seeking for participants in the upper and lower quartiles of multi-tasking activity (MMI).

Measure	Subcategory	Low MMI	High MMI	df	t-score	Cohen’s d
OSPAN		48.7 (16.0)	40.3 (16.3)	136	−3.04**	0.52
Perceived ability		59.2 (19.5)	69.1 (16.8)	136	3.21**	0.55
Impulsivity		1.96 (0.30)	2.11 (0.32)	135	2.79**	0.48
	Attentional	2.04 (0.41)	2.24 (0.46)	135	2.71**	0.46
	Motor	1.91 (0.32)	2.01 (0.35)	135	1.75	0.30
	Non planning	1.95 (0.39)	2.11 (0.41)	135	2.28*	0.40
Sensation seeking		1.43 (0.18)	1.51 (0.17)	135	2.51**	0.43
	Boredom	1.23 (0.20)	1.28 (0.16)	135	1.44	0.28
	Disinhibition	1.32 (0.26)	1.47 (0.30)	135	3.52**	0.53
	Thrill Seeking	1.67 (0.28)	1.71 (0.29)	135	0.67	0.14
	Experience	1.49 (0.24)	1.55 (0.22)	135	1.50	0.26

Also indicated are the degrees of freedom, t-test scores, and the effect size estimate, Cohen’s d.

* Significant at .05 level.

** Significant at .01 level.

A similar analysis contrasting participants reporting high or low concurrent use of a cell phone while driving is presented in Table 3. Participants reporting high concurrent cell-phone use while driving (i.e., concurrent use of a cell phone often, nearly every time, or every time while driving) scored significantly lower on the OSPAN task and significantly higher in the disinhibition and thrill seeking sub-scales of sensation seeking than participants reporting lower cell phone use while driving. (i.e., never or rarely using a cell phone while driving). There was also a trend for high cell phone users to report higher perceived ability (*p* = .058). In each case, the difference between groups reflected a “medium” effect size (e.g., between.3 and.5) [42].

**Table 3:** Means and standard deviations (in parentheses) for multi-tasking ability (OSPAN), perceived multi-tasking ability, impulsivity, and sensation seeking for participants in the upper and lower quartiles of cell phone use while driving.

Measure	Subcategory	Low Cell	High Cell	df	t-score	Cohen’s d
OSPAN		45.4 (15.8)	38.8 (17.7)	142	−2.36*	0.40
Perceived ability		59.7 (16.9)	65.8 (21.1)	141	1.90	0.32
Impulsivity		2.07 (0.33)	2.07 (0.32)	142	−0.03	0.05
	Attentional	2.21 (0.47)	2.20 (0.31)	142	−0.08	0.03
	Motor	1.92 (0.31)	1.94 (0.33)	142	0.46	0.06
	Non planning	2.11 (0.46)	2.09 (0.46)	142	−0.30	0.04
Sensation seeking		1.43 (0.18)	1.50 (0.15)	142	2.38*	0.40
	Boredom	1.26 (0.19)	1.27 (0.16)	142	0.32	0.06
	Disinhibition	1.34 (0.25)	1.48 (0.29)	142	3.16**	0.52
	Thrill Seeking	1.61 (0.30)	1.73 (0.26)	142	2.47**	0.43
	Experience	1.51 (0.24)	1.51(0.21)	142	0.12	0.00

Also indicated are the degrees of freedom, t-test scores, and the effect size estimate, Cohen’s d.

* Significant at .05 level.

** Significant at .01 level.

In a final analysis, we used linear regression to determine the unique contributors to a) multi-tasking activity, and b) cell phone use while driving. Here, we report the analysis using extreme groups; note however, that a similar pattern was produced using continuous measures of multi-tasking activity and cell phone use while driving. Based upon the univariate analyses reported above, we included multi-tasking ability (OSPAN), perceived multi-tasking ability, attentional impulsivity, non-planning impulsivity, and the disinhibition component of sensation seeking in the regression analysis. The standardized beta coefficients for the two regression analyses are provided in Table 4. For multi-tasking activity, the regression was significant, *F*(1,5) = 9.5, *p*<.01, *R* = 0.50, *SE* = 0.44. Examination of the beta coefficients indicates that all but non-planning impulsivity were significant predictors of multi-tasking activity. For cell phone use while driving, the regression was significant, *F*(1,5) = 3.7, *p*<.01, *R* = 0.36, *SE* = 0.47. Examination of the beta coefficients indicates that perceived ability and the disinhibition component of sensation seeking were significant predictors of the propensity to use a cell phone behind the wheel of an automobile.

**Table 4:** Linear regression standardized Beta coefficients and corresponding t-scores for multi-tasking ability (OSPAN), perceived multi-tasking ability, attentional impulsivity, non-planning impulsivity, and disinhibition in predicting multi-tasking activity and cell phone use while driving.

	Multi-tasking Activity		Cell Phone & Driving	
	Beta	t-score	Beta	t-score
OSPAN	−0.246	−3.40**	−0.079	−1.01
Perceived ability	0.327	4.19**	0.214	2.58**
Attentional impulsivity	0.222	2.56**	0.002	0.02
Non-planning impulsivity	0.070	0.86	0.101	0.12
Disinhibition	0.170	2.15*	0.208	2.54**

Beta refers to the standardized coefficients.

* Significant at .05 level.

** Significant at .01 level.

## Discussion

The findings provide an understanding of who is most likely to engage in multi-tasking and who is most likely to talk on the cell phone while driving. In examining who multi-tasks, the study provides an initial understanding of why people multi-task.

The results indicate that the persons who chronically multi-task are not those who are the most capable of multi-tasking effectively. To the contrary, OSPAN task performance was negatively correlated with self-reported multi-tasking activity. Perhaps more alarmingly, OSPAN task performance was also negatively correlated with self-reported usage of cellular communications while driving. Thus, the persons who talk on the cell phone the most while driving appear to be those who are the least capable of multi-tasking. As we mentioned earlier, research has shown that cell phone use significantly impairs driving performance [43], [44] and that over 24% of all accidents and fatalities on U.S. highways may be caused by distracted drivers [33]. The negative relation between cellular communication while driving and multi-tasking ability appears to further bolster arguments for legislation limiting the use of cell phones while operating a motor vehicle.

Both media related multi-tasking and cell phone usage while driving were positively correlated with the perceived ability to multitask. Perceptions of the ability to multi-task were found to be badly inflated; in fact, the majority of participants judged themselves to be above average in the ability to multi-task. These estimations had little grounding in reality as perceived multi-tasking ability was not significantly correlated with actual multi-tasking ability as measured by the OSPAN task. Thus, it appears that the persons who are most likely to multi-task and most apt to use a cell phone while driving are those with the most inflated views of their abilities.

The lack of concordance between perceived and actual multitasking ability is not surprising given the well-documented flaws characterizing self-assessments [9]. Research has shown that the correspondence between judgments of personal abilities and traits, and actual abilities and behavior is especially weak when the task or domain is poorly defined [45], [46] and when there is an absence of immediate and objective performance feedback [47], [48]. The concept of multi-tasking may be somewhat nebulous to laypersons. Moreover, the proper standards for assessing multi-tasking ability are unclear and objective feedback about the relative efficacy of performance is rarely received. Consequently, self-assessments of this seemingly important and desirable personal ability may be highly susceptible to bias.

Two important and interrelated personality traits were predictive of multi-tasking activity in our study. High sensation seekers, particularly those scoring high in disinhibition, were more likely than low sensation seekers to report media related multi-tasking and cell phone use while driving. As mentioned above, the disinhibition component of sensation seeking is associated with “seeking release” or “disinhibited social behavior”. Impulsivity, both the attentional and non-planning components of impulsivity, was also significantly correlated with higher levels of multi-tasking. Across all analyses, multi-tasking was most strongly associated with attentional impulsiveness. Thus, the people who are most likely to multi-task appear to be those who have difficulty focusing attention or concentrating on a single task.

The findings are consistent with our conceptualization of the general determinants of multi-tasking. Generally, multiple tasks present greater opportunity for rewards than singular tasks. Consequently, individuals who are approach oriented and attracted to the higher potential rewards of multi-tasking may be especially motivated to engage in multiple tasks simultaneously. Thus, impulsivity was significantly correlated with higher levels of multi-tasking. Moreover, individuals who perceive their multi-tasking ability to be high and thus, who are likely to anticipate the greatest rewards from engaging in many tasks concurrently reported greater multi-tasking activity. Finally, multiple tasks generally afford greater stimulation and challenge than singular tasks. Hence, high sensation seekers tended to report greater multi-tasking activity than low sensation seekers.

People may refrain from multi-tasking because of the risks and costs associated with taking on too much. Individuals who are less sensitive or averse to the higher potential losses entailed by multiple tasks may be more apt to multi-task. Thus, individuals who perceive their multi-tasking ability to be high and who are likely to anticipate the lowest costs or losses from engaging in many tasks concurrently reported greater multi-tasking activity. Additionally, persons who react with limited consideration of the negative consequences – high impulsives – reported greater multi-tasking activity than low impulsives. Similarly, high sensation seekers, particularly those high in the disinhibition component, reportedly engage in greater multi-tasking than low sensation seekers. Research suggests that high disinhibitors show evidence of less excitability in response to threats or potential losses than low disinhibitors [49]. Others studies have shown that sensation seeking is generally associated with less negative appraisals of risky situations, and lower levels of fear and anxiety [24], [25]. Hence, insensitivity to risk may generally contribute to multi-tasking and the willingness of high sensation seekers to engage in multiple tasks simultaneously.

Finally, people may engage in multi-tasking because they are unable to block out distractions and focus on a singular task. Consequently, individuals who are limited in the ability to inhibit involvement in secondary activities may be especially likely to multi-task. Consistent with this analysis, impulsivity was significantly associated with greater multi-tasking activity. Moreover, multi-tasking was shown to be particularly high amongst impulsive individuals who act without thinking and who have difficulty regulating their attention. Finally, individuals who scored low on the OSPAN task and who presumably have lower working memory capacity and executive control were more likely to engage in multi-tasking than persons who scored high on the OSPAN task. These findings clearly suggest that multi-tasking is a matter of who is able to *not* multi-task as much as it is a matter of who is able to multi-task.

The results are consistent with previous research by Ophir et al. [8] who found that high chronic multi-taskers lack some of the basic cognitive skills necessary for effective multi-tasking. Our research extends this important work by providing more direct evidence that lower multi-tasking ability is associated with greater multi-tasking activity. Moreover, we extend the findings to an important applied domain and show that drivers who talk on the cell phone are not the most capable multi-taskers. Finally, the negative correlation between OSPAN task performance and multi-tasking activity provides direct evidence that deficits in working memory and executive functioning are associated with higher levels of multi-tasking.

The media multi-tasking index (MMI) developed by Ophir et al. [8] was originally developed to assess the multiple forms of media used simultaneously by respondents. It was not designed to be a broad measure of everyday multitasking activity. Nevertheless, because media use is ubiquitous, we believe that the level of usage of multiple media is likely to be a good indicator of multi-tasking in other behavioral domains. The significant correlation between the MMI and a common and well known form of multi-tasking behavior – the usage of cell phones while driving - increases our confidence that general multi-tasking activity was measured.

As expected, impulsivity was significantly correlated with multi-tasking activity as measured by the MMI. However, impulsivity was not correlated with reported cell phone use while driving. For many people, the usage of cell phones while driving may be a premeditated behavior in which the decision to talk or not talk on the cell phone is made beforehand. Thus, it may not typically be a behavior requiring situational impulse control.

Sensation seeking and impulsivity have long been recognized to be closely related personality constructs [22]. Thus, following previous research [39], scores on the Sensation Seeking Scale were significantly correlated with responding on the BIS-11 in our study (*r = *.31). However, sensation seeking takes different forms. In particular, scholars have distinguished impulsive sensation seeking from non-impulsive sensation seeking, in part, because the pursuit of novel, varied, complex and intense experiences is commonly planned and deliberate [50], [21]. Thus, sensation seeking and impulsivity were not perfectly aligned in their relation to multi-tasking activity in our study. Specifically, sensation seeking was significantly correlated with cellular communication while driving (*r* = .13) whereas impulsivity was not (*r* = .00). This suggests that the sensation seeking underlying some cell phone usage while driving may be relatively deliberate as opposed to impulsive.

One limitation of our findings is that they are entirely correlational. Obviously, this limits the conclusions that can be drawn about the causes of multi-tasking activity. Future research may need to use experimental designs to examine how the rewards and costs associated with taking on multiple tasks, and the controllability of secondary task engagement affects the likelihood of multi-tasking. We believe that the relation between perceived multi-tasking ability and multi-tasking activity is one that is particularly likely to be bidirectional. A vast body of research has shown that people often draw inferences about their attitudes [51], [52] and self [53] from their behavior. People who frequently multi-task may infer from their behavior that they like to multi-task and that they are relatively good at it. Thus, although perceived multi-tasking ability may increase the willingness to multi-task, multi-tasking activity may also affect perceptions of multi-tasking ability.

A final limitation of the study involves our measure of multi-tasking ability. Although the OSPAN task has traditionally been used to measure working memory capacity it clearly meets the criteria for multi-tasking in that it involves multiple tasks characterized by distinct goals, stimuli, transformations, and response outputs. Nevertheless, the OSPAN task has not been psychometrically validated as a multi-tasking instrument. It is possible that a different ability-activity pattern might emerge with different measures of multi-tasking ability (or multi-tasking activity) and it remains for future research to determine if the current findings generalize to other measures of multi-tasking.
